# Mdivi-1 Inhibits Astrocyte Activation and Astroglial Scar Formation and Enhances Axonal Regeneration after Spinal Cord Injury in Rats

**DOI:** 10.3389/fncel.2016.00241

**Published:** 2016-10-19

**Authors:** Gang Li, Yang Cao, Feifei Shen, Yangsong Wang, Liangjie Bai, Weidong Guo, Yunlong Bi, Gang Lv, Zhongkai Fan

**Affiliations:** ^1^Department of Orthopaedics, The First Affiliated Hospital, Jinzhou Medical UniversityJinzhou, China; ^2^Department of Pathology, College of Basic Medical Sciences, China Medical UniversityShenyang, China; ^3^Department of Orthopaedics, The First Affiliated Hospital, China Medical UniversityShenyang, China

**Keywords:** spinal cord injury, mitochondrial division inhibitor-1, astrocytes, astroglial scar, axonal regeneration

## Abstract

After spinal cord injury (SCI), astrocytes become hypertrophic, and proliferative, forming a dense network of astroglial processes at the site of the lesion. This constitutes a physical and biochemical barrier to axonal regeneration. Mitochondrial fission regulates cell cycle progression; inhibiting the cell cycle of astrocytes can reduce expression levels of axon growth-inhibitory molecules as well as astroglial scar formation after SCI. We therefore investigated how an inhibitor of mitochondrial fission, Mdivi-1, would affect astrocyte proliferation, astroglial scar formation, and axonal regeneration following SCI in rats. Western blot and immunofluorescent double-labeling showed that Mdivi-1 markedly reduced the expression of the astrocyte marker glial fibrillary acidic protein (GFAP), and a cell proliferation marker, proliferating cell nuclear antigen, in astrocytes 3 days after SCI. Moreover, Mdivi-1 decreased the expression of GFAP and neurocan, a chondroitin sulfate proteoglycan. Notably, immunofluorescent labeling and Nissl staining showed that Mdivi-1 elevated the production of growth-associated protein-43 and increased neuronal survival at 4 weeks after SCI. Finally, hematoxylin-eosin staining, and behavioral evaluation of motor function indicated that Mdivi-1 also reduced cavity formation and improved motor function 4 weeks after SCI. Our results confirm that Mdivi-1 promotes motor function after SCI, and indicate that inhibiting mitochondrial fission using Mdivi-1 can inhibit astrocyte activation and astroglial scar formation and contribute to axonal regeneration after SCI in rats.

## Introduction

Spinal cord injury (SCI) is a global medical problem. It often leads to very limited regeneration of damaged axons and subsequent permanent functional impairment, the exact mechanisms of which remain to be elucidated (Silver and Miller, 2004; Tuszynski and Steward, 2012). The lack of spontaneous anatomical and functional repair is due not merely to an intrinsic inability of the neuron to regenerate its injured axon, but also to the presence of an inhospitable local environment in the lesion site constituting a physical and biochemical barrier—the so-called glial scar—composed essentially of reactive astrocytes (Silver and Miller, 2004). Astrocytes are the major cell type in the spinal cord and provide a variety of critical supportive functions that establish and maintain neuronal homeostasis (Tian et al., 2006). However, after SCI, they become hypertrophic and proliferative, and form a physical barrier at the site of injury, which significantly impedes axonal regeneration (Lin et al., 2014). In addition, chondroitin sulfate proteoglycans (CSPGs) form a biochemical barrier which also plays a crucial part in regeneration failure (Yiu and He, 2006; Gervasi et al., 2008; Jefferson et al., 2011). Therefore, inhibition of astrocyte activation, astroglial scar formation and CSPG production would create a favorable environment for axonal regeneration.

Astrocytes undergo cell division following SCI. Therefore, a possible approach for promoting axonal regeneration after SCI would be by inhibiting the division of cells that contribute to glial scar formation or by reducing production and secretion of inhibitory molecules (Karimi-Abdolrezaee and Billakanti, 2012). Mitochondria are organelles that are essential for a diverse range of important cellular functions, including cell metabolism, growth, differentiation, survival, and programmed cell death (DiMauro and Schon, 2008; Green and Van Houten, 2011). Recently, mitochondria were demonstrated to undergo frequent fission, and fusion (Cao et al., 2013), which are integrated with cell cycle progression (Mitra et al., 2009; Qian et al., 2012). Dynamin-related protein 1 (Drp1)-mediated mitochondrial fission is required for the proper progression of the cell cycle phases following G1/S transition. Thus, inhibition of mitochondrial division might be an important way to inhibit cell mitosis. However, it is unclear whether this would inhibit astrocyte activation and astroglial scar formation after SCI.

Mitochondrial division inhibitor 1 (Mdivi-1) is the most effective pharmacological inhibitor of mitochondrial division (Tanaka and Youle, 2008). It can cross the blood—brain barrier, and its half-life is about 12 h (Cui et al., 2016). Notably, Mdivi-1 exerts a protective effect in SCI (Li G. et al., 2015), spinal cord ischemia—reperfusion injury (Liu et al., 2015), acute cerebral ischemic injury (Cui et al., 2016), and seizures (Xie et al., 2013), and can also inhibit the proliferation, invasion and metastasis of breast cancer (Zhao et al., 2013) and ovarian cancer (Wang et al., 2015). However, the physiological and pathological roles of Mdivi-1 in astrocyte proliferation and astroglial scar formation after SCI are not yet known. Therefore, in the present study, we investigate the effects of Mdivi-1 on astrocyte proliferation, neurocan production, astroglial scar formation, and axonal regeneration after SCI in rats.

## Materials and methods

### Animals and experimental design

Adult female Sprague—Dawley rats, weighing 250–300 g, were provided by the Experiment Animal Center of Jinzhou Medical University. All experimental procedures were approved by the Institutional Animal Care and Use Committee of Jinzhou Medical University. Rats were randomly divided into three groups: SCI (Li G. et al., 2015), SCI + Mdivi-1 (Park et al., 2011), and sham (T9–11 laminectomy only). In the Mdivi-1 group, the rats were injected with Mdivi-1 (25 mg/kg i.p.; Sigma-Aldrich, St. Louis, MO) immediately after SCI and every 24 h thereafter for 3 or 9 days (Lin et al., 2014; Cui et al., 2016). Rats in the sham and SCI groups received an equivalent volume of dimethyl sulfoxide. Each group was then equally and randomly divided into three subgroups for the following experiments: (A) motor function test; (B) Western blot; and (C) histology (double immunofluorescent labeling, hematoxylin-eosin staining, and Nissl staining). The rats in all groups were sacrificed 3 days or 4 weeks after injury or laminectomy.

### SCI model establishment

Rats were anesthetized using pentobarbital sodium (40 mg/kg, i.p.). The weight-drop model of SCI was established as described previously (Li G. et al., 2015). In brief, the skin and muscle overlying the spinal column were incised and a laminectomy was performed at T9–11, leaving the dura intact. A 20 g weight was then dropped onto the exposed T10 region of the spinal cord from a height of 25.0 mm. Postoperatively, all animals received an injection of 0.9% normal saline (30 ml/kg) to prevent postoperative dehydration. Rats were housed individually in cages under a 12 h light/dark cycle, with free access to food and water, and underwent assisted urination three times per day after SCI throughout the study.

### Motor function evaluation

The motor function of rats in each group was evaluated using the Basso, Beattie, and Bresnahan (BBB) scale at 3 days and 4 weeks after SCI, as described previously (Li G. et al., 2015). The experiments were performed six independent times.

### Western blot

Spinal cords were removed 3 days or 4 weeks after SCI. The protein lysates were fractioned on 6 or 10% SDS-polyacrylamide gels, and then transferred to 0.45 μm polyvinylidene fluoride membranes. The membranes were blocked with 0.1% bovine serum albumin for 1 h, then incubated overnight at 4°C with primary antibodies against glial fibrillary acidic protein GFAP (ab7260, 1:2000), neurocan (ab31979, 1:300), proliferating cell nuclear antigen (PCNA; ab29, 1:1000), growth-associated protein-43 (GAP-43; ab16053, 1:1000) (all from Abcam, USA), and β-actin (C4; sc-47778, 1:1000; Santa Cruz Biotechnology, Inc., USA). The membranes were then incubated with the corresponding second antibody: goat anti-rabbit or anti-mouse IgG-HRP (sc-2004 or sc-2005; 1:2000; Santa Cruz Biotechnology) at room temperature for 1 h. The remaining steps were performed as described in our previous study (Li G. et al., 2015), and the protein bands were analyzed using NIH Image J software. Grayscale values of bands corresponding to GFAP, PCNA, neurocan, and GAP-43 were normalized to that of β-actin to determine expression levels of the proteins of interest. The experiments were performed six independent times.

### Immunofluorescent double labeling

At 3 days and 4 weeks after surgery, the injured spinal cords from rats in all groups were fixed in 4% paraformaldehyde, immersed in 30% sucrose, embedded in compound 4583, and then sectioned on a freezing microtome as described previously (Li G. et al., 2015). Serial 5-μm-thick transverse frozen sections from the epicenter of the SCI were used for immunofluorescent double labeling and hematoxylin-eosin staining. After permeabilizing the cells and blocking non-specific binding sites, these spinal cord tissue sections were incubated with the following primary antibodies diluted in 5% normal goat serum (005-000-121, Jackson): anti-GFAP (ab7260, 1:500), anti-neurocan (ab31979, 1:50), anti-PCNA (ab29, 1:200), anti-GAP-43 (ab16053, 1:200) (all from Abcam, USA), and anti-NeuN (MAB377X, 1:50; Millipore, USA) in a humidified chamber overnight at 4°C. Tissue sections were incubated with the secondary antibodies Alexa Fluor 488 goat anti-rabbit IgG (H+L; A-11034, 1:250; Thermo Fisher Scientific, USA) and Alexa Fluor 594 goat anti-mouse IgG (H+L; A-11005, 1:250; Thermo Fisher Scientific) for 1 h at room temperature in the dark. After immunostaining, the sections were incubated with 1 μg/ml 4′,6-diamidino-2-phenylindole (DAPI) for 5 min in the dark to mark the nuclei. Negative control sections were incubated with phosphate-buffered saline in place of the primary antibody. The sections were visualized under a fluorescent microscope (Leica DMI4000B, Germany) connected to an Olympus Magnafire digital camera (Olympus Corp., Melville, NY, USA). Each fluorescent color was photographed under the same fluorescence microscopy settings. To avoid counting the same cell in more than one section, we counted every 11th section (100 μm apart). Immunopositive cells (those showing red or green fluorescence) were counted using the counting function in Photoshop CS3, and expressed as a percentage of the total number of cells (those showing blue fluorescence) (Li G. et al., 2015). Mean fluorescence intensity was used to quantify neurocan and GAP-43 expression, measured using Image J. Mean fluorescence intensity = Integrated Density/Area (Tian et al., 2006). The experiments were performed six independent times.

### Nissl staining

The 5 μm transverse frozen sections were dried and then soaked overnight in a 1:1 mixture of alcohol and chloroform in the dark at 22 ± 1°C. The following day, the sections were rehydrated, and stained in 0.1% cresyl violet solution (Sigma, St. Louis, MO, USA) for 5 min. Differentiation, dehydration, and rinsing was performed as described previously (Li H.-T. et al., 2015). The sections were then mounted with Permount (Beyotime Institute of Biotechnology, Shanghai, China) and observed under a light microscope (Olympus) equipped with a CCD camera (Leica DMI4000B, Germany). Surviving neurons were counted using Photoshop CS3. The experiments were performed six independent times.

### Hematoxylin-eosin staining

To measure the cavity area of spinal cord tissue in each group after SCI, animals in all groups were sacrificed 4 weeks after injury. Spinal cord tissue was cut into frozen sagittal sections of 5 μm thickness. Spinal cord tissue was prepared, cut into frozen sagittal sections of 5 μm thickness, and stained with hematoxylin-eosin, as described previously (Tian et al., 2006) and examined with a light microscope (Olympus) equipped with a CCD camera (Leica DMI4000B). The maximum area of the cavity in the sagittal sections including the damage epicenter was measured in Photoshop CS3, using the area analysis function. The experiments were performed six independent times.

### Statistical analysis

All data are expressed as mean ± standard deviation (SD), and were analyzed using one-way analysis of variance (ANOVA) with the least significant difference (LSD) *post hoc* test. SPSS version 19.0 was used for all analysis. *P* < 0.05 was considered statistically significant.

## Results

### Mdivi-1 inhibits astroglial proliferation at 3 days after SCI

**Figure 1:**
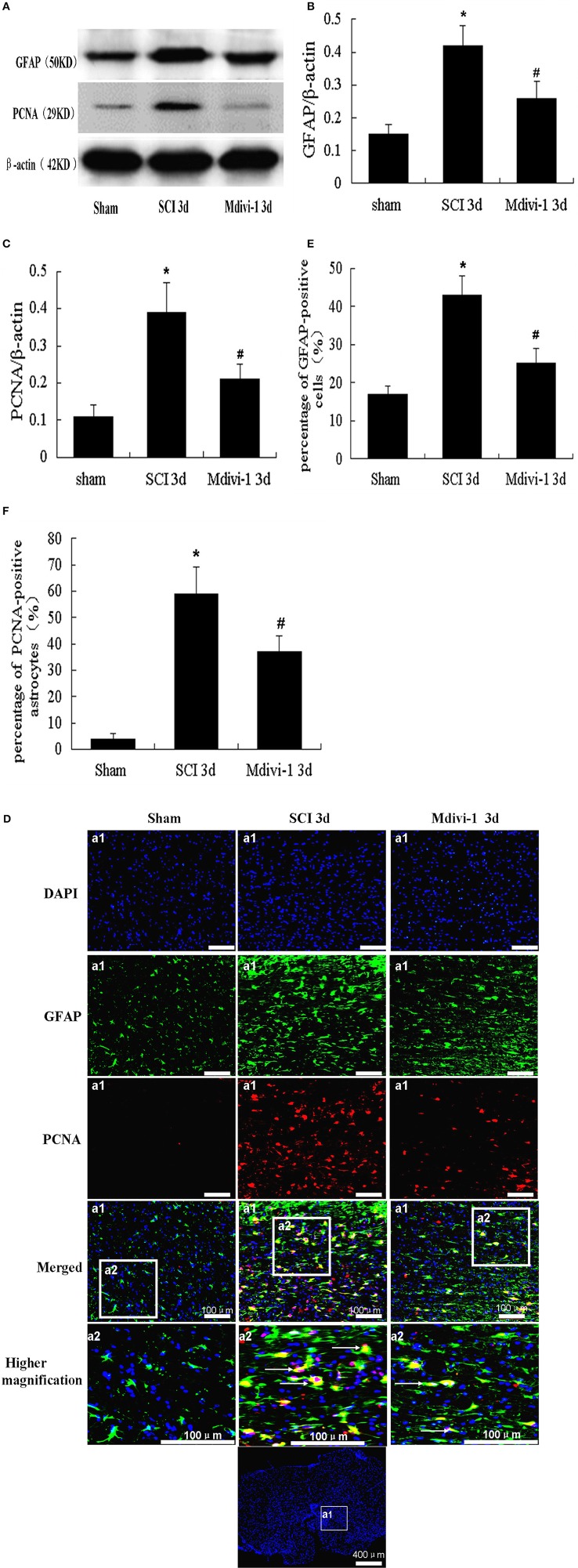
**Mdivi-1 increases the expressions of GFAP and PCNA in astrocytes at 3 days after SCI. (A–C)** Western blot analysis and quantitative analysis of GFAP **(A,B)** and PCNA **(A,C)** (*n* = 6, respectively). **(D–F)** PCNA/GFAP/DAPI triple labeling and quantitative analysis of GFAP **(D,E)** and PCNA **(D,F)** in astrocytes (*n* = 6, respectively). White arrows indicate proliferative astrocytes. The expression of PCNA and GFAP, and the number of GFAP-positive cells and PCNA-positive astrocytes, were greater in the SCI group than in the sham group. PCNA and GFAP expression, and the number of GFAP-positive cells and PCNA-positive astrocytes were lower in the Mdivi-1 group than in the SCI group. ^*^P < 0.01 vs. Sham. ^#^*P* < 0.01 vs. SCI.

Astrocytes upregulate the expression of GFAP, a widely recognized astrocyte marker, and form a physical barrier at the site of injury that impedes axonal regeneration (Lin et al., 2014). PCNA is known to be a reliable marker of proliferating cells (Tian et al., 2006). We used western blots to investigate astroglial proliferation after Mdivi-1 treatment. GFAP and PCNA expression were significantly greater in the SCI group (0.42 ± 0.08 and 0.40 ± 0.06, respectively) than in the sham group (0.15 ± 0.05 and 0.11 ± 0.03), indicating that astroglial proliferation was induced after SCI (*P* < 0.01) (Figures 1A–C). In the Mdivi-1 group (0.26 ± 0.06 and 0.21 ± 0.06), GFAP and PCNA expression were lower than in the SCI group which suggests that Mdivi-1 inhibits astroglial proliferation (*P* < 0.01).

Double immunofluorescence labeling of PCNA/GFAP was performed to confirm astrocyte proliferation, in combination with the nuclear marker DAPI. Thus, proliferative astrocytes were visible as PCNA/GFAP/DAPI-positive cells, showing red dots (PCNA) in a green cells (GFAP) with a blue nucleus (DAPI). Not all GFAP-positive cells were positive for PCNA, and some GFAP-negative cells were positive for PCNA (Figures 1D–F). There were more GFAP-positive cells and PCNA-positive astrocytes in the SCI group (0.43 ± 0.06 and 0.59 ± 0.11, respectively) than in the sham group (0.17 ± 0.03 and 0.04 ± 0.02). However, there were fewer GFAP-positive cells and PCNA-positive astrocytes in the Mdivi-1 group (0.25 ± 0.05 and 0.36 ± 0.05) than in the SCI group (*P* < 0.01). These findings indicate that inhibition of mitochondrial division by Mdivi-1 can inhibit astroglial proliferation after SCI.

### Mdivi-1 decreases the expressions of GFAP and neurocan at 4 weeks after SCI

**Figure 2:**
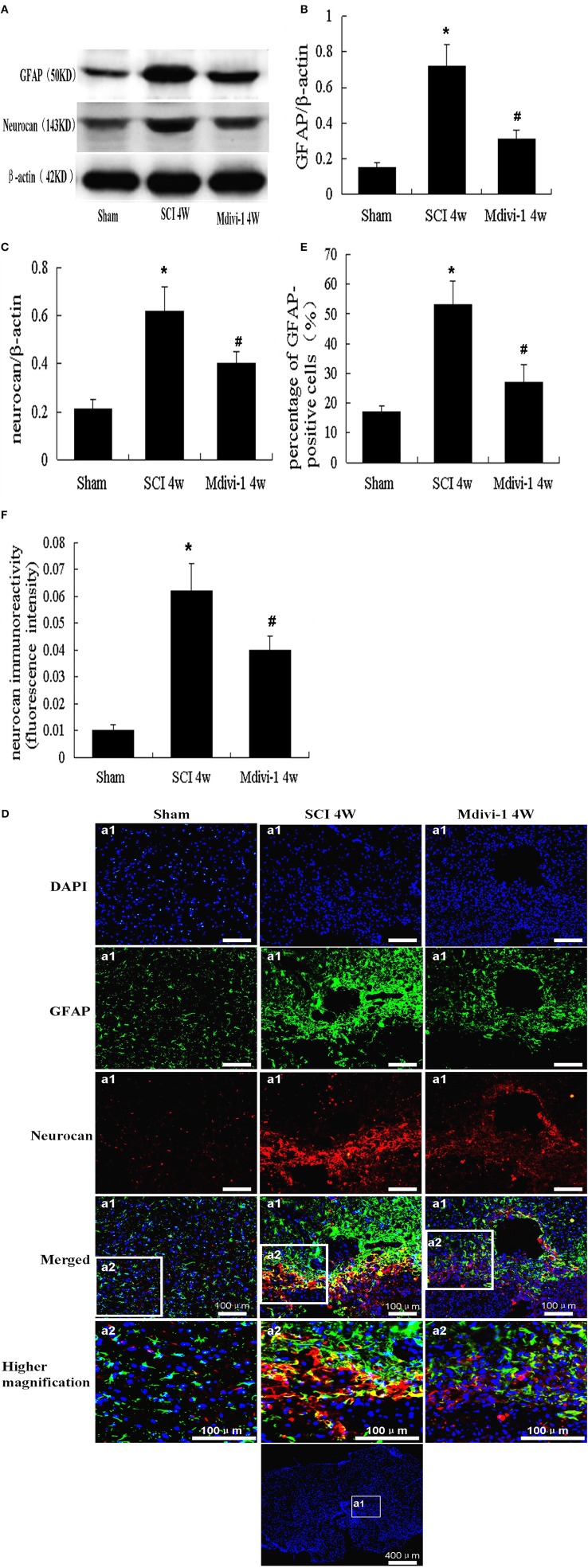
**Mdivi-1 decreases the expressions of GFAP and neurocan at 4 weeks after SCI. (A–C)** Western blot analysis and quantitative analysis of GFAP **(A,B)** and neurocan **(A,C)** (*n* = 6, respectively). **(D–F)** Neurocan/GFAP/DAPI triple labeling and quantitative analysis of GFAP **(D,E)** and neurocan **(D,F)** in astrocytes (*n* = 6, respectively). Compared with the sham group, the expression of GFAP and neurocan, number of GFAP-positive cells, and mean fluorescence intensity of neurocan were elevated after SCI. GFAP, neurocan expression, number of GFAP-positive cells, and mean fluorescence intensity of neurocan were lower in the Mdivi-1 group than in the SCI group. ^*^P < 0.01 vs. Sham. ^#^*P* < 0.01 vs. SCI.

Neurocan is a CSPG produced mainly by astrocytes (Jones et al., 2003; Silver and Miller, 2004). We used Western blots to investigate neurocan expression after SCI with or without Mdivi-1 treatment. GFAP and neurocan expression in the SCI group (0.72 ± 0.12 and 0.62 ± 0.10, respectively) were significantly greater than in the sham group (0.15 ± 0.04 and 0.21 ± 0.04; *P* < 0.01). However, expression of GFAP and neurocan in the Mdivi-1 group (0.33 ± 0.05 and 0.40 ± 0.08, respectively) was lower than in the SCI group. This suggests that Mdivi-1 inhibits neurocan production after SCI (*P* < 0.01), which might reduce the biochemical barrier to axonal regeneration (Figures 2A–C).

Double immunofluorescence labeling for neurocan and GFAP was performed to further characterize the production and secretion of neurocan in astrocytes after SCI with or without Mdivi-1 treatment. Triple-positive neurocan/GFAP/DAPI-positive cells were observed. Neurocan (red dots) was distributed mainly in the extracellular matrix and cytoplasm of astrocytes (green); nuclei appeared blue. Expression of GFAP and neurocan was lower in the SCI group (0.54 ± 0.10 and 0.064 ± 0.012, respectively) than in the sham group (0.17 ± 0.02 and 0.011 ± 0.003; *P* < 0.01). Furthermore, in the Mdivi-1 group, the number of GFAP-positive cells and mean fluorescence intensity of neurocan (0.26 ± 0.08 and 0.040 ± 0.007, respectively) were lower than in the SCI group (*P* < 0.01). These findings indicate that astrocyte proliferation and neurocan expression and secretion were inhibited by Mdivi-1 after SCI (Figures 2D–F).

### Mdivi-1 increases the expressions of GAP-43 at 4 weeks after SCI

**Figure 3:**
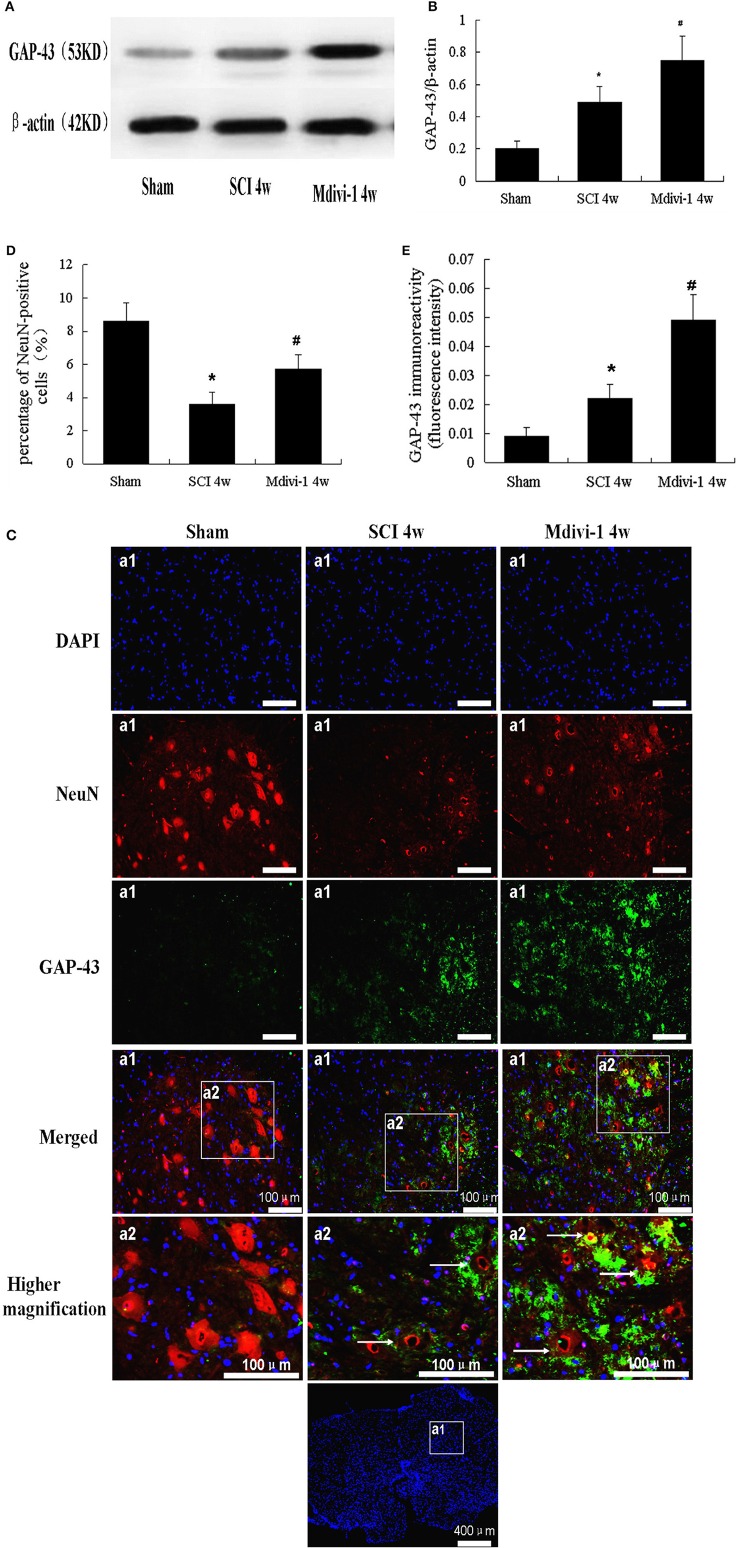
**Mdivi-1 increases the expressions of GAP-43 at 4 weeks after SCI. (A,B)** Western blot analysis **(A)** and quantitative analysis of GAP-43 **(B)** (*n* = 6, respectively). (**C–E)** Cells triple-labeled for GAP-43/NeuN/DAPI and quantification of NeuN **(C,D)** and GAP-43 **(C,E)**. White arrows: neurons indicate GAP-43. Expression of GAP-43 was greater in the SCI group than in the sham group, and the percentage of neurons was lower. Compared with the SCI group, Mdivi-1 can increase GAP-43 expression and the percentage of surviving neurons. ^*^P < 0.01 vs. sham. ^#^*P* < 0.01 vs. SCI.

GAP-43 is a cytoplasmic and membrane-associated protein located mainly in neuronal growth cones and upregulated within growing neurites (Benowitz and Routtenberg, 1997; Irwin and Madsen, 1997). Western blots were used to investigate GAP-43 expression after SCI with or without Mdivi-1 treatment. Compared with the sham group (0.20 ± 0.06), the expression of GAP-43 was significantly greater after SCI (0.50 ± 0.09; *P* < 0.01). Moreover, GAP-43 expression was greater in the Mdivi-1 group (0.75 ± 0.15) than in the SCI group (*P* < 0.01), which suggests that Mdivi-1 enhances axonal regeneration after SCI (Figures 3A,B).

Immunofluorescent staining of GAP-43 was performed to further identify it in neurons after Mdivi-1 treatment. Higher magnification indicated that the GAP-43/NeuN/DAPI triple-labeled neurons had green, punctate GAP-43 dots in red neurons with a blue nucleus. The percentage of neurons and mean fluorescence intensity of GAP-43 were calculated. Not all neurons were positive for GAP-43 (Figure 3C). In the SCI group, the percentage of neurons (3.6 ± 0.7) was lower than in the sham group (8.6 ± 1.1), whereas the mean fluorescence intensity of GAP-43 was greater (0.022 ± 0.005) than in the sham group (0.009 ± 0.003; *P* < 0.01). Compared with the SCI group, the percentage of neurons and GAP-43 expression were both higher in the Mdivi-1 group (5.7 ± 0.9 and 0.049 ± 0.009; *P* < 0.01). These findings provide evidence that axonal regeneration and neuronal survival were enhanced by Mdivi-1 after SCI (Figures 3C–E).

### Mdivi-1 decreases the number of surviving neurons at 4 weeks after SCI

**Figure 4:**
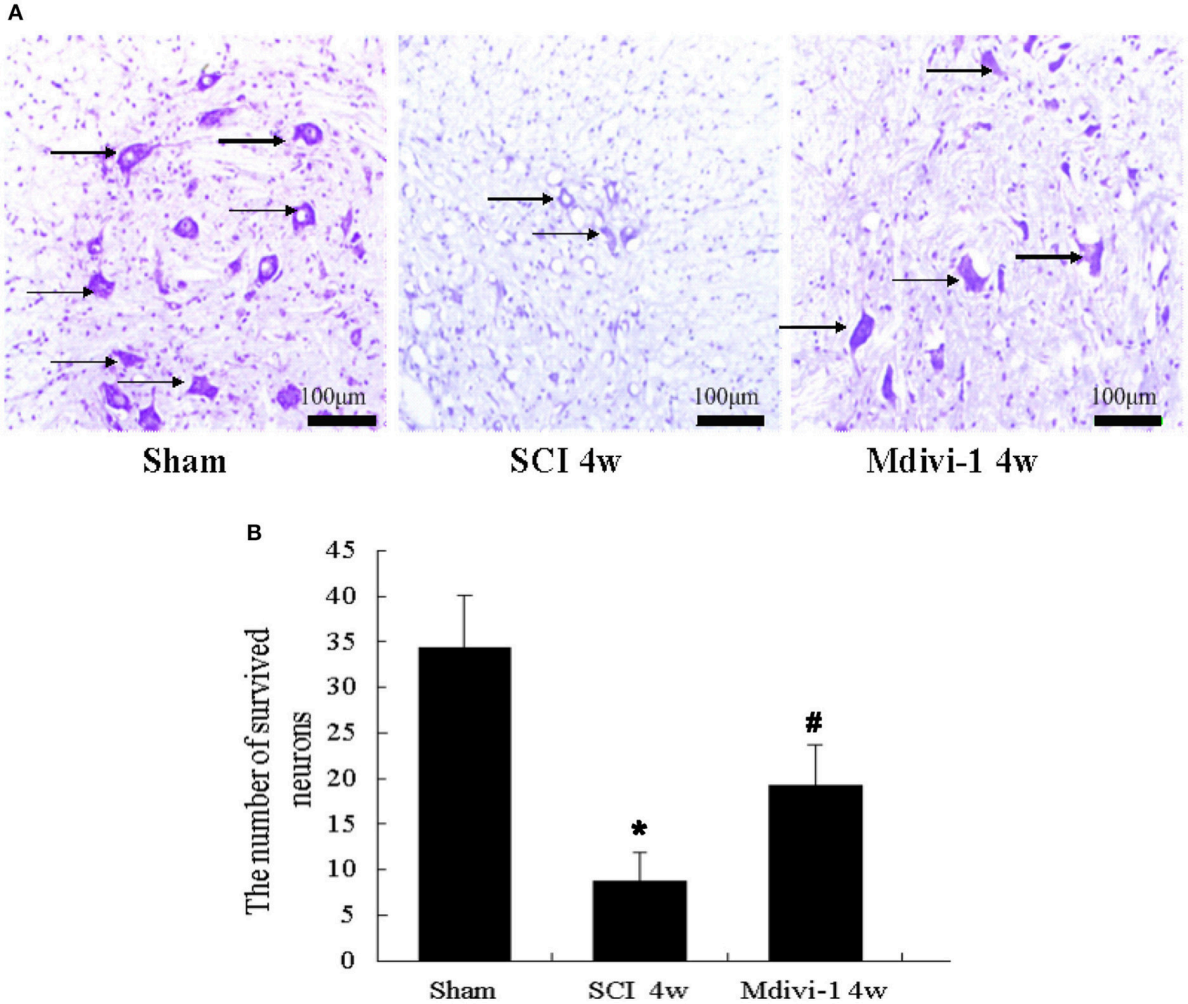
**Mdivi-1 decreases the number of surviving neurons at 4 weeks after SCI. (A)** Histological assessments of spinal cord tissue. Black arrows: surviving neurons. **(B)** Quantification of surviving neurons. The number of surviving neurons was lower in the SCI group than in the sham group. Compared with the SCI group, the number of surviving neurons was lower in the Mdivi-1 group. ^*^P < 0.01 vs. sham. ^#^*P* < 0.01 vs. SCI.

To determine the effects of Mdivi-1 on spinal cord tissue, Nissl staining was used to illustrate the injured areas in the different groups. The surviving neurons in the sections were subsequently examined at a higher magnification. The number of surviving neurons was lower in the SCI group than in the sham group (*P* < 0.01); however, the number of surviving neurons was greater in the Mdivi-1 group than in the SCI group (*P* < 0.01). These results demonstrate the significant protective effect of Mdivi-1 (Figure 4).

### Mdivi-1 decreases the cavity formation at 4 weeks after SCI

**Figure 5:**
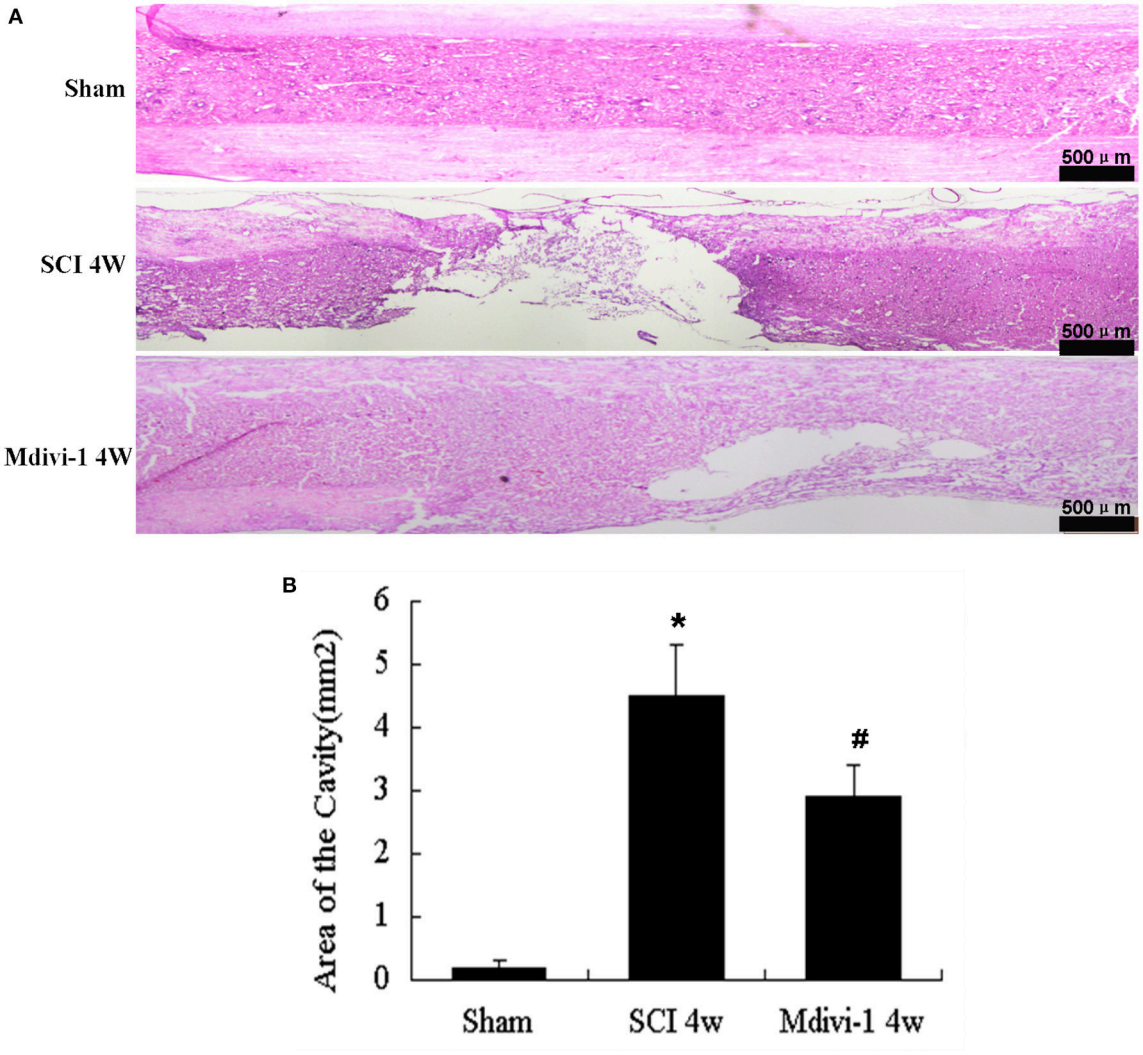
**Mdivi-1 decreases the cavity formation at 4 weeks after SCI. (A)** Representative hematoxylin-eosin staining micrographs show cavity formation in each group after SCI. **(B)** Quantification of cavity area shows that Mdivi-1-treated animals had significantly smaller spinal cord cavities compared than those in the SCI group. ^*^P < 0.01 vs. Sham. ^#^*P* < 0.01 vs. SCI.

The size of the cavity in the spinal cord can reflect astroglial scar formation after SCI (Tian et al., 2006). Compared with the sham group (0.20 ± 0.10 mm^2^), the cavity area was significantly greater after SCI (4.5 ± 0.8 mm^2^, *P* < 0.01). Compared with the SCI group, the cavity area was smaller in the Mdivi-1 group (2.9 ± 0.6 mm^2^, *P* < 0.01), which suggests that Mdivi-1 inhibits astroglial scar formation after SCI (Figure 5).

### Mdivi-1 improves the hindlimb motor function at 3 days and 4 weeks after SCI

**Figure 6:**
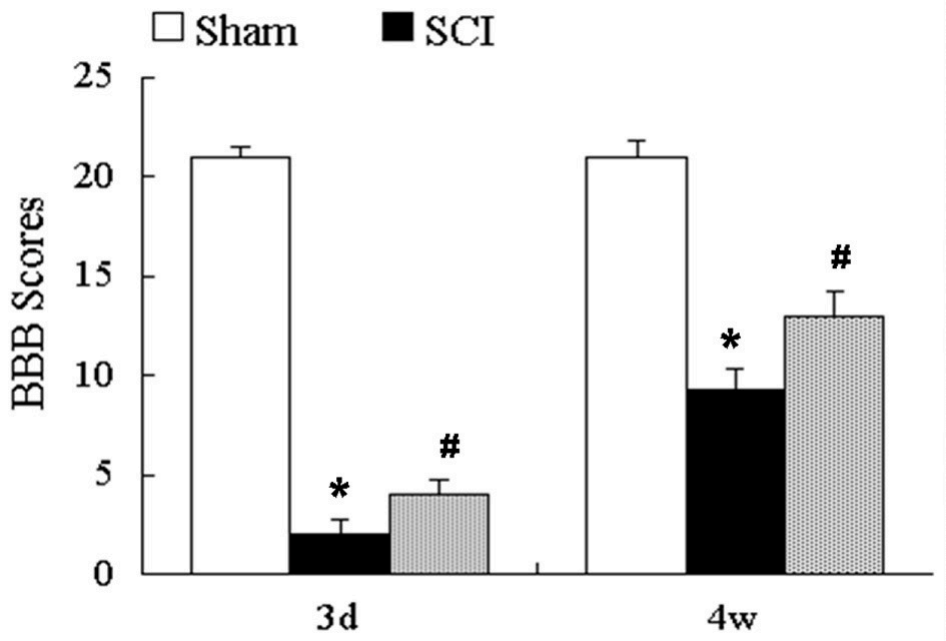
**Mdivi-1 improves the hindlimb motor function at 3 days and 4 weeks after SCI**. BBB scores showed that Mdivi-1 reduced hindlimb motor dysfunction at 3 days and 4 weeks after SCI. ^*^P < 0.01 vs. Sham. ^#^*P* < 0.01 vs. SCI.

The BBB locomotor rating scores were assessed at 3 days and 4 weeks after SCI. Compared with the sham group (3 days, 20.6 ± 0.6; 4 weeks, 20.6 ± 0.6), the BBB scores were significantly lower after SCI (2.0 ± 0.8 and 9.3 ± 1.1, respectively; *P* < 0.01). Compared with the SCI group, the BBB scores were significantly greater in the Mdivi-1 group at 3 days and 4 weeks (4.0 ± 0.8 and 13 ± 1.3, respectively; *P* < 0.01). These findings support the neuroprotective effect of Mdivi-1 (Figure 6).

## Discussion

Astrocytes are the most abundant glial cells in the central nervous system (CNS). In chronic SCI, they become hypertrophic and proliferative (Wilhelmsson et al., 2006), upregulate the production of inhibitory CSPG (Bradbury et al., 2002; Su et al., 2011), and eventually contribute to the formation of an astroglial scar that might hinder axonal regeneration in chronic SCI (Lin et al., 2014). Finding effective ways to inhibit glial scar formation and enhance axon regeneration is essential after SCI. To our knowledge, this study is the first study to confirm that Mdivi-1 inhibits astrocyte activation and astroglial scar formation, and contributes to axonal regeneration as well as recovery of motor function after SCI in rats.

Cell cycle regulation is a relatively new research direction in nerve repair therapy (Zhu et al., 2007). Restarting of the cell cycle plays a key role in astroglial activation and proliferation after SCI (Byrnes et al., 2007; Zhu et al., 2007; Lin et al., 2014). Administration of olomoucine, flavopiridol, and MEK can decrease neuronal cell death and inhibit glial proliferation after SCI. These agents reduce axon growth-inhibitory molecule expression and astroglial scar formation at the site of injury by inhibiting the cell cycle *in vivo* (Tian et al., 2006; Byrnes et al., 2007; Li et al., 2011; Wu et al., 2011; Lin et al., 2014). Moreover, cell cycle inhibition protects against traumatic brain injury by reducing glial proliferation and scar formation in rats (Di Giovanni et al., 2005). Results from an astrocyte scratch-wound model showed that scratch damage can significantly promote the proliferation of cultured astrocytes and increase the number of 5-bromo-deoxyuridine-positive cells *in vitro* (Yang et al., 2009). Cyclins and PCNA expression increased significantly after SCI (Tian et al., 2006), suggesting that astrocytes proliferate and then form a dense glial scar in the damaged area, thus preventing normal reconstruction of axons and functional recovery after SCI *in vivo*. Previous studies found that PCNA expression peaked 3 days after SCI (Tian et al., 2006), and that changes in glial scars and motor function reached a plateau in rats at 4 weeks after SCI (Hu et al., 2010; Li et al., 2011). So we chose 3 days and 4 weeks after SCI as our evaluation points. Western blot and immunofluorescence double labeling showed that the expression of PCNA increased significantly at 3 days after SCI in rats, which is consistent with previous studies (Tian et al., 2006). Notably, Mdivi-1 significantly inhibited the expression of PCNA at 3 days after SCI in rats, suggesting that inhibiting mitochondrial division after SCI can inhibit the cell cycle progression of astrocytes, and supporting previous studies (Mitra et al., 2009; Qian et al., 2012). Surprisingly, we also found some PCNA-immunoreactivity in GFAP-negative cells that might be microglia or endothelial cells, which further supports previous studies (Krum and Khaibullina, 2003; Tian et al., 2007).

Following SCI, astrocytes are activated and show hypertrophy of the cell body, abnormal proliferation, and upregulation of GFAP (Tang et al., 2003; Bramanti et al., 2010). They rapidly secrete a variety of inhibitory proteins, especially CSPGs, and ultimately form the glial scar tissue in the SCI lesion, which is the physical and biochemical barrier of repair, and is not conducive to the growth or regeneration of axons (Tang et al., 2003; Yuan and He, 2013). In addition to an increase in astrocyte number at the lesion site in response to SCI injury, GFAP production, a feature of astrogliosis, also increases, and its production can reflect the changes in number or activation degree of astrocytes (Jones et al., 2003). Our present Western blot and immunofluorescence studies show that astrocytes undergo changes in morphology and GFAP expression at 3 days and 4 weeks after SCI, which supports previous studies (Yang et al., 2009; Bramanti et al., 2010). Importantly, Mdivi-1 significantly inhibited the expression of GFAP and astrocyte performance after SCI, suggesting that inhibition of mitochondrial division can inhibit astroglial proliferation and hypertrophy after SCI.

CSPGs are extracellular matrix molecules that are widely expressed throughout the developing and adult CNS (Bradbury et al., 2002). *In vitro* studies demonstrate their potential to restrict neurite outgrowth, and it is believed that CSPGs also inhibit axonal regeneration after CNS injury *in vivo* (Yuan and He, 2013). After SCI, members of the CSPG family, including neurocan, brevican, versican and NG2, are upregulated, and might have important roles in limiting axonal regeneration (Jones et al., 2003; Tang et al., 2003). Previous studies have reported that neurocan, secreted by reactive astrocytes, increases within 2 days in injured spinal cord, peaks at 2 weeks, and remains persistently elevated for 4 weeks after SCI (Bradbury et al., 2002; Jones et al., 2003). Our Western blot and immunofluorescence results showed that neurocan expression was significantly increased 4 weeks after SCI, whereas Mdivi-1 inhibited the expression of neurocan significantly. This indicates that inhibition of mitochondrial fission may inhibit astrocyte proliferation, then inhibit the expression, and secretion of neurocan, and finally inhibit the formation of biochemical barriers after SCI. Moreover, results of the hematoxylin-eosin stain show that Mdivi-1 significantly reduces cavity formation after SCI, suggesting that inhibition of mitochondrial fission can also inhibit the formation of the physical glial barrier after SCI. These results support previous studies (Byrnes et al., 2007; Zhu et al., 2007; DiMauro and Schon, 2008; Lin et al., 2014) and contribute to our understanding of the relationship between mitochondrial fission and astroglial proliferation after SCI.

Our previous results indicate that Mdivi-1 plays a role in reducing apoptosis by inhibiting the translocation of dynamin-related protein 1 (Drp1) and Bax to the mitochondria (Li G. et al., 2015). At the same time, Liu et al. (2015) reported that Mdivi-1 may be an effective therapeutic agent for spinal cord ischemia–reperfusion injury via activation of large-conductance Ca^2+^-activated K^+^ channels as well as reduction of oxidative stress, mitochondrial dysfunction and neuronal apoptosis. To further confirm the protective effect of Mdivi-1 *in vivo*, we investigated the survival of neurons directly by immunoïňĆuorescence staining and Nissl staining. Spinal cord neurons were marked by NeuN (the neuronal marker). The percentage of neurons via immunoïňĆuorescence staining and the number of surviving neurons via Nissl staining decreased significantly at 4 weeks after SCI and increased in the Mdivi-1 group, which supports our previous study. In the present study, we found that Mdivi-1 can inhibit astroglial proliferation and increase the number of surviving neurons, but the relationship between these two results needs further study. In addition, the upregulation of GAP-43 is considered a useful marker of neurite growth or sprouting after SCI (Benowitz and Routtenberg, 1997; Irwin and Madsen, 1997). Notably, in this study, we found that SCI alone led to a limited upregulation of GAP-43 expression. Moreover, the Mdivi-1-treated group had a significantly higher expression of GAP-43 at 4 weeks after SCI, which may be related to the promotion of neuronal survival. However, we cannot rule out the possibility that the increase in GAP-43 by Mdivi-1 is related to the inhibition of astroglial scar formation (Lin et al., 2014). We also found that Mdivi-1 significantly mitigated functional deficits as assessed by the measures of the BBB scale. Our data indicated that Mdivi-1 administration has a protective effect after SCI, which supports previous studies (Li G. et al., 2015; Liu et al., 2015).

In cancer growth, mitochondria grow continuously throughout the cell cycle, and the organization of the mitochondrial network is sophisticatedly controlled across the different phases of the cell cycle (Qian et al., 2013). At the G1/S border, mitochondria form a single, giant tubular network, which is associated with increased energy production in order to prepare for the initiation of the highly energy consuming process—DNA synthesis (Mitra et al., 2009). In addition, such mitochondrial hyperfusion also facilitates the mixing of mitochondrial contents such as mtDNA between adjacent mitochondria, to maintain a homogenous mitochondrial network within the cell, thus ensuring proper inheritance following cellular division. The formation of a highly connected mitochondrial network at G1/S border is transient. During the subsequent S, G2 and M phases, the hyperfused mitochondrial network is then disassembled and becomes increasingly fragmented (Qian et al., 2013). Notably, mitochondrial fission disorders can lead to sustained mitochondrial hyperfusion beyond the G1/S border, which further inhibits mitosis and ultimately cell proliferation (Qian et al., 2012). Our results indicate that Mdivi-1 inhibits astrocyte activation after SCI. However, whether the effects of Mdivi-1 on the astroglial phenotype result from the inhibition of mitochondrial fission in astrocytes directly, or indirectly via Mdivi's protective effect against neuronal apoptosis, warrants further investigation. We will examine the detailed mechanism of Drp1-mediated mitochondrial fission during astroglial division using gene knockout and transfection technology, and confirm whether Mdivi-1 can inhibit mitochondrial fission in astroglia in the astrocyte scratch-wound model.

In summary, this is the first study to demonstrate that Mdivi-1 inhibits astrocyte activation and astroglial scar formation, and contributes to the axonal regeneration after SCI in rats. This indicates that regulation of mitochondrial dynamics maybe an effective therapeutic target for SCI. However, as the effect of Mdivi-1 is not cell specific, we cannot rule out the possibility that neuroprotection by Mdivi-1 is associated with the inhibition of other proliferating cells, such as microglia and endothelial cells, in the spinal cord after SCI. Further research is needed to clarify these issues.

## Author contributions

GLv and ZF designed and supervised the project. GLi, FS, YW, LB, WG, and YB performed research; YC analyzed data and GLi wrote the article.

### Conflict of interest statement

The authors declare that the research was conducted in the absence of any commercial or financial relationships that could be construed as a potential conflict of interest.
